# Exogenous GA_3_ Application Enhances Xylem Development and Induces the Expression of Secondary Wall Biosynthesis Related Genes in *Betula platyphylla*

**DOI:** 10.3390/ijms160922960

**Published:** 2015-09-23

**Authors:** Huiyan Guo, Yucheng Wang, Huizi Liu, Ping Hu, Yuanyuan Jia, Chunrui Zhang, Yanmin Wang, Shan Gu, Chuanping Yang, Chao Wang

**Affiliations:** 1 State Key Laboratory of Tree Genetics and Breeding, Northeast Forestry University, 26 Hexing Road, Harbin 150040, China; E-Mails: wsghy1307@126.com (H.G.); wangyucheng1029@126.com (Y.W.); LHZ249450439@126.com (H.L.); hupingmojiezuo@aliyun.com (P.H.); jiayuayua2015@126.com (Y.J.); zcrlwzy@126.com (C.Z.); wangyanmin1919@aliyun.com (Y.W.); 2 Department of Life Science and Technology, Mudanjiang Normal College, Mudanjiang 157012, China; E-Mail: gushan0731@126.com; 3 Forestry Science Research Institute of Heilongjiang Province, Harbin 150081, China

**Keywords:** *Betula platyphylla* SUK, GA_3_, seed germination, xylem development, secondary wall biosynthesis related genes

## Abstract

Gibberellin (GA) is a key signal molecule inducing differentiation of tracheary elements, fibers, and xylogenesis. However the molecular mechanisms underlying the effect of GA on xylem elongation and secondary wall development in tree species remain to be determined. In this study, *Betula platyphylla* (birch) seeds were treated with 300 ppm GA_3_ and/or 300 ppm paclobutrazol (PAC), seed germination was recorded, and transverse sections of hypocotyls were stained with toluidine blue; the two-month-old seedlings were treated with 50 μM GA_3_ and/or 50 μM PAC, transverse sections of seedling stems were stained using phloroglucinol–HCl, and secondary wall biosynthesis related genes expression was analyzed by real-time quantitative PCR. Results indicated that germination percentage, energy and time of seeds, hypocotyl height and seedling fresh weight were enhanced by GA_3_, and reduced by PAC; the xylem development was wider in GA_3_-treated plants than in the control; the expression of NAC and MYB transcription factors, *CESA*, *PAL*, and *GA* oxidase was up-regulated during GA_3_ treatment, suggesting their role in GA_3_-induced xylem development in the birch. Our results suggest that GA_3_ induces the expression of secondary wall biosynthesis related genes to trigger xylogenesis in the birch plants.

## 1. Introduction

The expansion of xylem tissue is crucial for plant growth, particularly the growth of woody plants. Therefore, this process is of particular interest to commercial forestry. Unraveling the signaling processes mediating xylem activation and secondary wall development in tree species may shed new light on potential mechanisms to maximize crop yield. During primary growth, the procambium differentiates into vascular tissues including xylem, first forming protoxylem, and vascular cambium develops through secondary growth to form secondary xylem [1]. The present study showed that these processes are controlled by plant hormone regulators including auxin, cytokinin, gibberellins, and ethylene [2].

Gibberellins (GAs) are tetracyclic diterpenoid plant growth hormones that regulate diverse physiological processes including seed germination, stem elongation, leaf expansion, root growth, and the development of reproductive organs [3,4,5]. GA_3_ induces the germination of dormant *Avena fatua* caryopses via regulation of ABA and ROS-antioxidant status [6]. The seed germination is faster and higher than in control seeds under endogenous GA_3_ treatment in macaw palm [7]. GA signaling in the cambium has been reported to mediate xylogenesis, and promote fiber elongation [8]. Supplementation with exogenous GA significantly increased the ratio of xylem area to total plant area and increased the proportion of fibers in *Arabidopsis* [9]. The increase in endogenous GA_3_ content induces significant increases of xylogenesis in tobacco [10]. Overexpression of the GA receptors PttGID1.1 or PttGID1.3 in aspen stimulates cambial activity to enhance xylem production [8]. The role of signaling processes in the activation of xylem and secondary wall development requires investigation.

It was hypothesized that signals, such as GA, activate the gene-regulated transcriptional network [11]. A previous study found that NTL8 (a membrane-bound NAC transcription factor) mediated the salt regulation of seed germination via GA metabolism in *Arabidopsis* [12]. A recent study indicated that *SbMYB2* and *SbMYB7* might regulate secondary biosynthesis by GA pathway in transgenic tobacco plants [13]. Transcription of *CESA*, *PAL*, and other genes related to cellulose, xylan, and lignin biosynthesis is regulated by the transcription factors NAC and MYB [14,15,16]. Therefore, we hypothesized that the *NAC*, *MYB*, *CESA*, and *PAL* genes of birch plants also controlled the xylem development process regulated by GA.

In this study, in order to investigate the effect of GA_3_ on the germination of seeds and development of xylem in the birch we monitored the germination, hypocotyl height, and fresh weight of seedlings, measured transverse sections of hypocotyls and seedling stems and analyzed the expression of NAC and MYB transcription factors, *CESA*, *PAL* and *GA* oxidase genes by real-time quantitative PCR (RT-PCR). This work further characterizes the mechanisms of xylem development in birch.

## 2. Results and Discussion

### 2.1. Germination of Birch Seeds

Open pollinated white birch *(B. platyphylla* SUK) seeds were moistened with GA_3_ and/or paclobutrazol (PAC), an inhibitor of GA_3_ biosynthesis, and water as a control.

**Figure 1:**
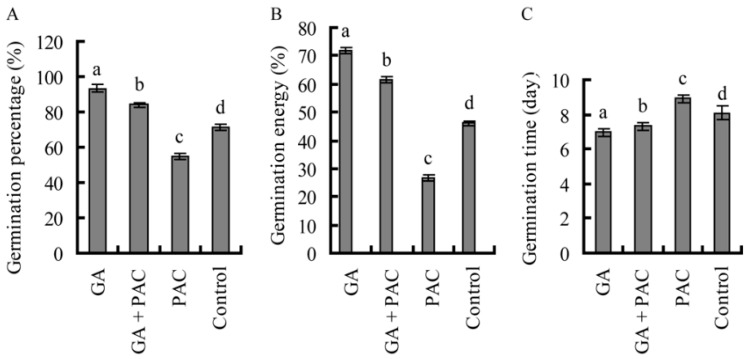

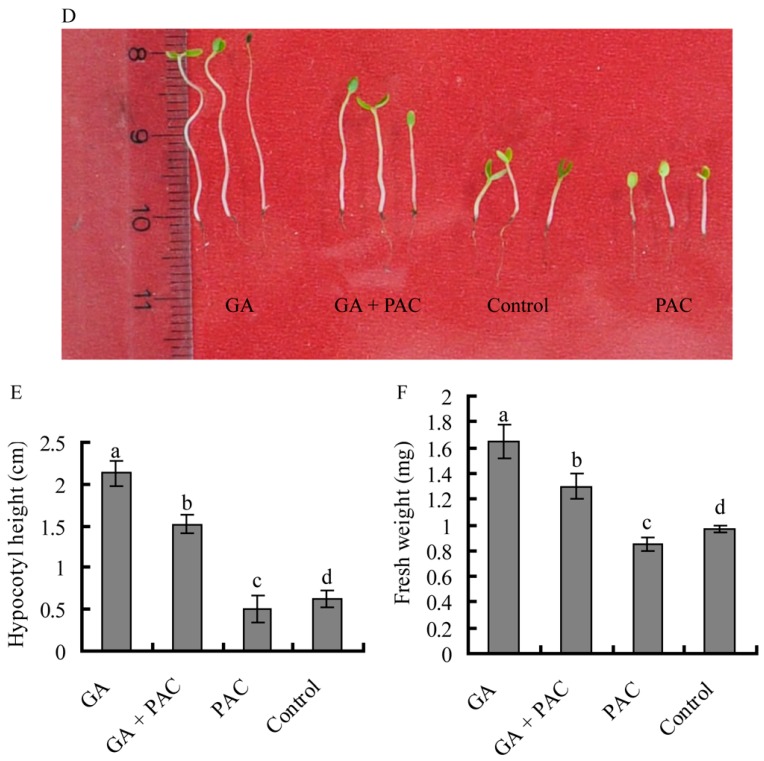
The germination of birch seeds treated with GA_3_ and/or paclobutrazol (PAC). (**A**) Germination percentage (%) of birch seeds treated with GA_3_ and/or PAC; (**B**) Germination energy (%) of birch seeds treated with GA_3_ and/or PAC; (**C**) Germination time (day) of birch seeds treated with GA_3_ and/or PAC; (**D**) Growth of birch seedlings; (**E**) Hypocotyl height of birch seedling; and (**F**) Fresh weight of birch seedlings. Lower case letter indicates *p* < 0.05.

Germination was enhanced by GA_3_ and repressed by PAC. A significantly higher percentage of GA_3_-treated seeds germinated (93.33%) than control seeds (71.33%), PAC-treated (54.67%) or GA_3_ + PAC-treated seeds (84.00%) (Figure 1A). The germination energy of GA_3_-treated seeds (72.00%) was also higher than control seeds (46.00%), PAC-treated (26.67%) or GA_3_ + PAC-treated seeds (61.33%) (Figure 1B). GA_3_-treated seeds also germinated faster (6.95 days) than control (8.08 days) or PAC-treated seeds (8.91 days); GA_3_ + PAC-treated seeds also germinated after roughly seven days (Figure 1C). Seed dormancy and germination are regulated by a number of genes and environmental factors [17]. Various methods have been used to terminate seed dormancy, including hormonal, light and/or temperature treatments [18,19]. Endogenous GAs have previously been used to terminate dormancy and promote seed germination [20]. Our results showed that GA_3_ promoted germination of birch seeds and ameliorated the impact of PAC on germination.

Application of GA_3_ significantly induced elongation of birch seedling hypocotyl within the first 15 days of germination (Figure 1D–F). The mean hypocotyl treated with GA_3_ was 2.13 cm in length and longer than the control hypocotyls, which measured 0.62 cm (Figure 1D,E). The fresh weight of seedlings treated with GA_3_ was 1.65 mg, which was also higher than that of control seedlings weighing 0.97 mg (Figure 1F). At 0.5 cm, the hypocotyls of seeds treated with PAC were more stunted than control hypocotyls; the fresh weight of these seedlings was 0.85 mg, which was lower than control seedlings (Figure 1D–F), suggesting that the application of PAC had a negative effect on birch seedling hypocotyl growth. However, the growth inhibition induced by PAC was ameliorated by application of GA_3_, the mean hypocotyl length and the fresh weight of seedlings treated with GA_3_ + PAC, respectively, were 1.52 cm and 1.30 mg, further suggesting that GA_3_ accelerated hypocotyl elongation and seedling growth, consistent with previous studies [21,22].

### 2.2. The Primary Xylem Development of Birch Hypocotyls

**Figure 2:**
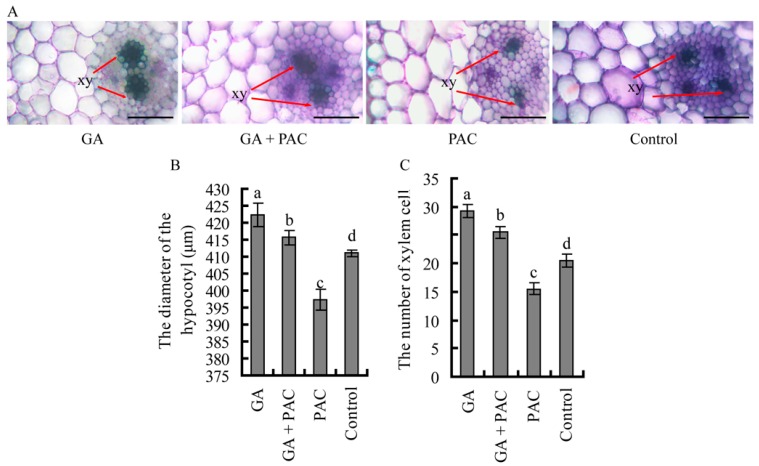
Transverse sections of 15-day-old birch seedlings germinating under GA_3_ and/or PAC. The hypocotyl base was sectioned 15 days after germination. (**A**) Toluidine blue staining analysis of the differentiation of xylem. xy, xylem cell. Bars = 50 μm; (**B**) The diameter of the hypocotyl (μm); and (**C**) The number of xylem cell. Lower case letter indicates *p* < 0.05.

The hypocotyl base was sectioned 15 days after germination and stained with toluidine blue (Figure 2A). Xylem differentiation was observed in hypocotyls treated with GA_3_ and GA_3_ + PAC. However, differentiation of the primary xylem was weaker in control hypocotyls or hypocotyls treated with PAC than in those treated with GA_3_ or GA_3_ + PAC. The diameter of the hypocotyl in GA_3_ or GA_3_ + PAC was wider than that in control; however, it was narrower in PAC than in control (Figure 2B); the number of xylem cell (Figure 2C) in GA_3_ (about 29) or GA_3_ + PAC (about 26) was more than that in control (about 21), PAC (about 16) was the smallest. These results indicate that GA promotes xylem differentiation and primary xylem development.

### 2.3. Growth and Secondary Xylem Development of Birch Seedlings

**Figure 3:**
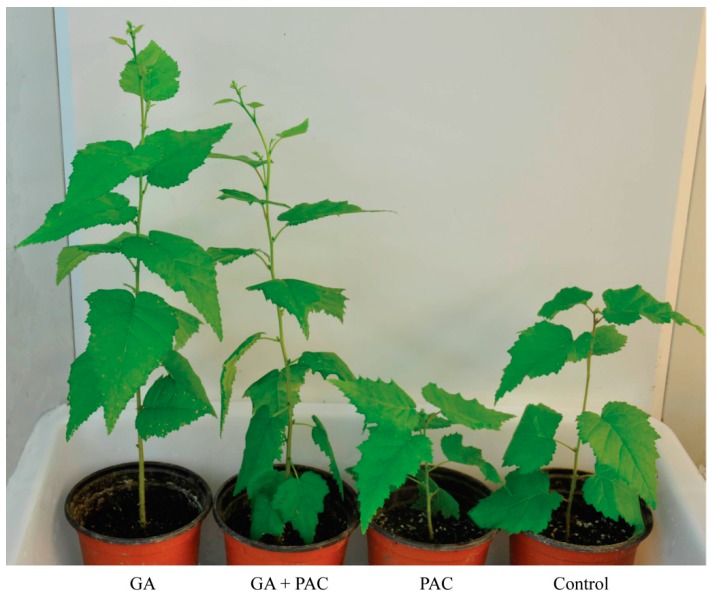
Phenotypic changes of 2-month-old birch seedlings under GA_3_ and/or PAC.

Two-month-old birch seedlings were moistened with GA_3_ and/or PAC. We found that GA_3_ and/or PAC had a similar effect on the growth of birch seedlings as in birch hypocotyls. Seedlings treated with GA_3_ (36.97 cm) or GA_3_ + PAC (33.90 cm) were taller with active apical growth than seedlings treated with water (21.83 cm) or PAC (11.03 cm) (Figure 3), in which the apical shoot and apical growth were significantly slower, suggesting that GA_3_ also promotes stem elongation and apical growth.

**Figure 4:**
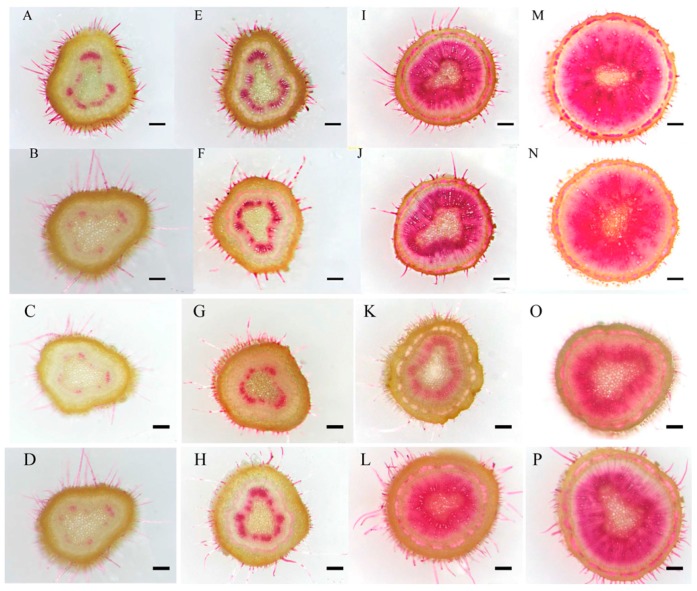

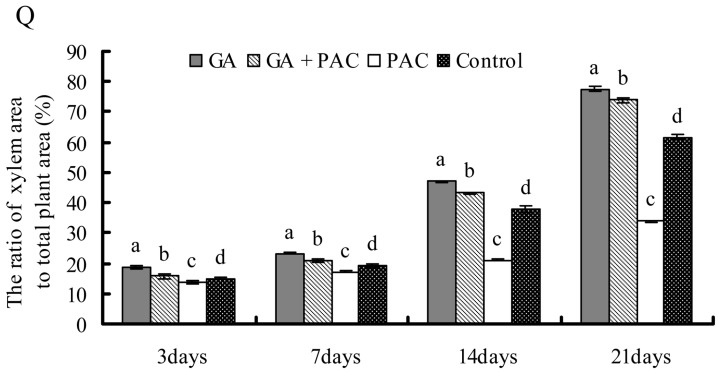
Transverse sections of 2-month-old birch seedlings and the ratio of xylem area to total area under GA_3_ and/or PAC treatments. (**A**–**D**) GA and/or PAC treatment for 3 days; (**E**–**H**) GA and/or PAC treatment for 7 days; (**I**–**L**) GA and/or PAC treatment for 14 days; and (**M**–**P**) GA and/or PAC treatment for 21 days. (**A**,**E**,**I**,**M**) GA treatment; (**B**,**F**,**J**,**N**) GA + PAC treatment; (**C**,**G**,**K**,**O**) PAC treatment; and (**D**,**H**,**L**,**P**) water treatment (control). Phloroglucinol–HCl was used to stain lignin to highlight xylem vessels and fibers. Bars = 1 mm; (**Q**) The ratio of xylem area to total area in birch plants under GA_3_, GA_3_ + PAC, PAC and control for 3, 7, 14 or 21 days. Error bars were obtained from multiple replicates of the real-time PCR. Lower case letter indicates *p* < 0.05.

After different GA and/or PAC treatments time of two-month-old birch seedling, transverse sections of stems were obtained and stained with phloroglucinol–HCl to highlight xylem tissue. During development, the area of xylem in the birch stem expanded, and expanded most rapidly in seedlings treated with GA_3_ or GA_3_ + PAC; seedlings treated with PAC alone developed less quickly than seedlings treated with water alone (Figure 4A–P). The ratio of xylem area to total plant area under GA_3_, GA_3_ + PAC, PAC and control was calculated, respectively (Figure 4Q). The ratio of xylem area to total plant area was slightly higher under GA_3_ than control and PAC treatments, which were about 18.5% and 23.3% in GA_3_, 15% and 19.2% in control, and 13.6% and 17.1% in PAC, respectively at 3 and 7 day. The ratio of xylem area to total area in plants was significantly higher under GA_3_ treatment compared with under control condition, but was significantly lower under PAC treatment at 14 and 21 day. The ratio was 47.1% in GA_3_, 37.9% in control and 20.9% in PAC at 14 day, and 77.5% in GA_3_, 61.7% in control and 40% in PAC at 21 day.

The GA_3_ pathway promotes xylogenesis in many species [23,24]. In *Fraxinus mandshurica* Rupr. *var. japonica* Maxim. seedlings, the width of xylem in stems was enhanced by GAs (GA_3_ and GA_4_) [23]. In *Zinnia elegans*, a slight increase in the frequency of tracheary element (TE) differentiation and a substantial increase in lignin content were observed under GA_3_ treatment [23]. PAC treatment probably inhibited xylem development by inhibiting GA synthesis, as the growth deficiency was rescued by exogenous application of GA_3_ [4]. Our results (Figure 4) further demonstrate that GA_3_ is an important factor in regulating xylem development of birch plants, consistent with previous studies.

### 2.4. Gene Selection and Expression Pattern under GA_3_ Treatment

In a previous study [25], differential gene expression (DGE) indicated that 4 *NAC*, 5 *MYB*, 2 *CESA*, and 2 *PAL* genes were altered with higher RPKM values among tension wood (TW), opposite wood (OW) and normal wood (NW), suggesting that these genes may play a role in regulating xylem development in the birch. Therefore, these potential secondary cell wall (SCW) genes were selected for expression analysis in this study.

**Figure 5:**
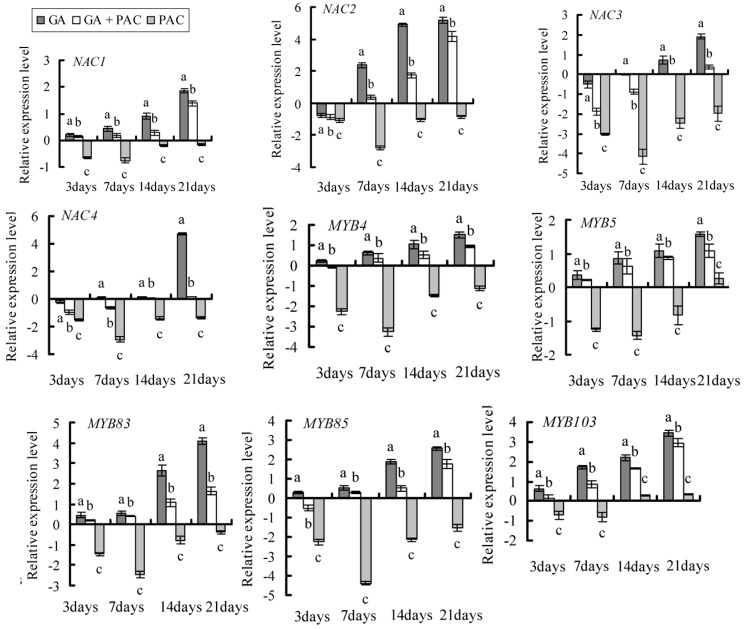

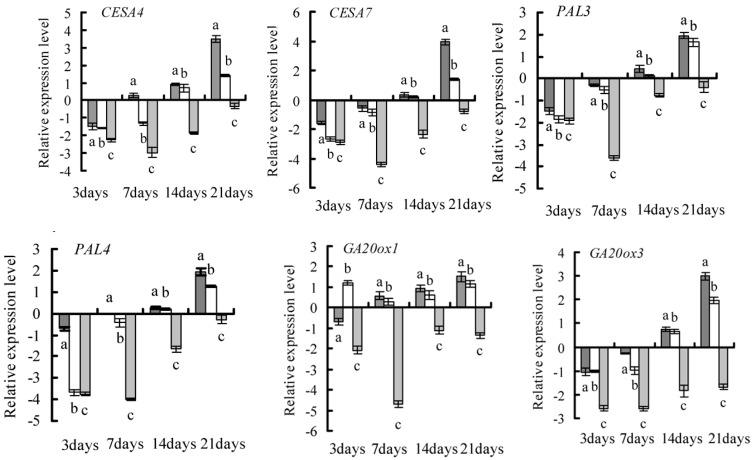
Expression analysis of the genes in birch plants under GA_3_ and/or PAC. These genes include *NAC*, *MYB*, *PAL*, *CESA*, and *GA20ox*. Error bars were obtained from multiple replicates of the real-time PCR. Lower case letter indicates *p* < 0.05.

The expression of 4 *NAC*, 5 *MYB*, 2 *CESA*, 2 *PAL*, and 2 *GA20ox* genes related to xylem development obtained using the DGE and bioinformatics analysis in seedlings treated with GA_3_ and/or PAC was analyzed by real-time quantitative PCR. The expression of most genes was up-regulated on days 7, 14, and 21 in plants treated with GA_3_ or GA_3_ + PAC; the expression of almost all genes was down-regulated in plants treated with PAC, on days 7, 14, and 21 compared with plants treated with water alone (Figure 5).

Differentiation of vascular cambium into xylem mother cells is regulated by plant hormones. The coordinated activation of secondary wall biosynthesis genes during wood formation is mediated by a transcriptional network encompassing secondary wall NAC and MYB master switches and their downstream transcription factors [26]. Our results suggest that GA_3_ induced the expression of 4 NAC and 5 MYB transcription factors, and the 2 *CESA*, 2 *PAL*, and 2 *GAoxidase* genes, suggesting that birch SCW transcription factors respond to GA_3_. Genes, such as *NAC2*, *CESA4*, *PAL3*, *GA20ox1*, and *GA20ox3*, were down-regulated at 3 day in plants treated with GA_3_ or GA_3_ + PAC compared with control. However, these genes were up-regulated during the treatment period resulting in altered xylem phenotype, indicating that the treatments were long enough to modify the phenotypes and affect the genes expression via direct or indirect pathway. The temporary decrease and subsequent increase of gene expression suggests complex molecular mechanisms of gene regulation.

NAC transcription factors are the key regulators of secondary wall synthesis. Specific loss of secondary walls was observed at the valve margins of *Arabidopsis* in NST1 mutants, indicating that NST1 mediated secondary wall biosynthesis [27]. GA_3_ treatment of birch seedlings up-regulated the expression of *BplNAC1* and *BplNAC2* (Figure 5). Previous reports suggested that *NAC* genes were up-regulated by some hormones [28]. OsNAC2 affected plant height by regulating the GA pathway in rice [29]. In this study, xylem development was promoted by GA_3_, and expression of *BplNAC1* and *BplNAC2* genes, which are closely related to *SND2* gene (*AtNAC073*) and *NST1* gene (*AtNAC043*) of *Arabidopsis*, were up-regulated by GA_3_. AtNAC073 belongs to the SND2 family, related to genes involved in secondary cell wall development and promotion of fiber cell area [30]. Taken together, our results indicate that BplNAC1 and BplNAC2 are involved in xylem development, respond to the GA signal pathway, and were induced by GA_3_.

In general, the MYB transcription factors downstream of SND1, are regulated by SND1. We found that BplMYB83 is similar to AtMYB46 and AtMYB83, and BplMYB103 is similar to AtMYB103 of *Arabidopsis* in protein sequence. Both *MYB* genes of birch were induced by GA_3_, but suppressed by PAC during birch seedling growth (Figure 5). AtMYB46 and AtMYB83 are directly activated by SND1 and act as molecular switches in the SND1-mediated transcriptional network during secondary wall deposition [31,32]. AtMYB103 was previously reported to regulate secondary cell wall formation [33]. The expression of *GAMYB* was induced by GA, and is a component of the GA-responsive pathway, leading to GA-inducible gene expression during seed germination in barley [34]. We found that GA_3_ promoted xylem development and that *BplMYB83*, *BplMYB5*, and *BplMYB52* were up-regulated by GA_3_, suggesting that these genes promoted secondary xylem development in birch.

NAC and MYB transcription factors regulate the biosynthesis of secondary cell wall components including cellulose and lignin. MYB is reported to bind promoters of cellulose and lignin synthesis genes, such as *CESAs* and *PAL*, and induce their expression [35,36]. In this study, *BplCESA4* and *BplCESA7* were up-regulated in GA_3_-treated seedlings. CESAs are critical to xylem development and are necessary for synthesis of secondary cell wall in plants [37,38]. BplCESA4 and BplCESA7 share homology with AtCESA4, and AtCESA7, which were implicated in secondary cell wall biosynthesis in *Arabidopsi*s [39,40]. The DGE and sequence analysis suggested that BplCESA4 and BplCESA7 may be associated with xylem development and deposition of secondary cell wall in birch. Germination and embryonic axis growth in *Medicago truncatula* are inhibited by ABA, and the expression of the *CESAs* and other cell-wall loosening and expansion genes were also down-regulated by ABA treatment [41]. In *Eucalyptus tereticornis*, all three EtCESA transcripts were also up-regulated by GA [42], suggesting that *CESA* expression is influenced by hormones. In this study, GA promotion of hypocotyl and xylem expansion may be attributed to the induction of genes involved in cell wall biosynthesis, including CESAs. The expression of *BplPAL3* and *BplPAL4* was also up-regulated by GA_3_. BplPAL3 has sequence similarity with AtPAL1 and AtPAL2. Based on analysis of the EST data, the AtPALs are likely candidates for lignification, or overlapping PAL metabolic networks, which are operative in vascular tissues [43]. These results suggest that the two birch *PAL* genes were involved in GA-induced birch xylem development.

BplGA20ox1 and BplGA20ox3 are homologous to AtGA20ox of *Arabidopsis*. GA 20-oxidase is an enzyme that catalyzes the last three steps in the synthesis of active GAs and is a potential control point in the regulation of GA biosynthesis [44]. GA20ox is also induced by many hormones [45]. *AtGA20ox1* and *AtGA20ox2* promote hypocotyl and internode elongation [46]. *PtGA20ox* was recently reported to be highly expressed in the mature xylem of vascular tissues, and directly mediates secondary xylem formation [47]. In this study, the expression of birch *BplGA20ox1* was inhibited by PAC, resulting in shorter stem and slower growth. GA_3_ induced longer stems and faster growth. A previous study found that *GA20ox2* transcription was noticeably reduced following GA application within 15 min of hormone treatment in *ga1-3* mutant *Arabidopsis* [48]; The expression of *AtGA20ox2* and *AtGA20ox3* was up-regulated initially, and then down-regulated by exogenous GA4 in *ga1-3* seeds [49]. In our study, the expression of *GA20ox1* and *GA20ox3* was transiently down-regulated, and then up-regulated, which may be due to the fact that the treatments were long enough and further implied that the *GA20ox* mediates the endogeous GA content regulation at an early stage and promotes the growth and development of birch through the direct or indirect pathway.

## 3. Experimental Section

### 3.1. Seed Germination and Treatment

Open pollinated Northeast White Birch (*B. platyphylla*) seeds were collected and stored in a sealed plastic box at 4 °C. The seeds were rinsed thoroughly with tap water prior to germination in plastic pots and subsequently incubated at 30 °C. Germinated seeds were moistened every second day with 300 ppm GA containing 0.1% (*v*/*v*) Tween-80 and 0.1% (*v*/*v*) ethanol and/or 300 ppm PAC (an inhibitor of GA biosynthesis), or water containing 0.1% (*v*/*v*) Tween-80 and 0.1% (*v*/*v*) ethanol as control. After seven days, only water was used to moisten all seeds. Fifty seeds were incubated under each experimental condition, and all experiments were carried out in triplicate. The number of germinated seeds was recorded every day for 15 days and seeds were considered germinated when the emerging radicle was approximately 2 mm long. On day 15 the percentage of germinated seeds (germination percentage), germination energy, and time to germination (germination time) were calculated when no further germination occurred for three days in all treatments. Hypocotyl height was recorded and fresh seedlings were also weighed on day 15 after germination. The hypocotyls of germinated seeds were collected and fixed in FAA solution (50% ethanol, 5% glacial acetic acid, and 5% formaldehyde) for anatomical analysis.

(1) $$\text{Germination Percentage}=\frac{\text{the Number of Germinated Seeds}}{\text{the Number of Total Seeds}}\times 100\%$$

(2) $$\text{Germination Energy}=\frac{\text{the Number of Germinated Seeds to Peak}}{\text{the Number of Total Seeds}}\times 100\%$$

(3) $$\text{Germination Time}=\frac{\sum (G_{i}\times D_{i})}{\sum G_{i}}$$

*G_i_* represents the number of germinated seeds on day *i*, and *D_i_* denotes the number of days taken for germination.

### 3.2. Seedling Growth and Treatment

Birch seeds were sown in plastic pots, and incubated under a 16/8 h day/night (24/22 °C) at a 60%–70% relative humidity and 400 μmol·m^−2^·s^−1^ light intensity in a greenhouse. Two months later, seedlings grew in pots that had achieved the same height and were sprayed every second day with 50 μM GA_3_ and/or 50 μM PAC solution or water for 21 days. The stems of birch seedlings were harvested separately on days 3, 7, 14, or 21 after GA_3_ and/or PAC treatment. Six seedlings were used under each treatment at each harvest time, and three biological replicates were used for each experiment (in total 18 seedlings per condition). The basal stems of three seedlings in each condition were fixed in FAA solution (70% ethanol, 5% glacial acetic acid, and 5% formaldehyde) and used for anatomical analysis. The stems of the other three seedlings were immediately frozen in liquid nitrogen and stored at −80 °C for RNA extraction, which was then used for RT-PCR analysis.

### 3.3. Histological Analysis

To visualize xylem development in hypocotyls (at 15 days post-germination) and birch seeding stems (at least two months post-germination), the bases of hypocotyls were cut into about 50-µm sections by hand. The fifth internodes from base to tip of the two-month-old birch stems after 21 days treatment under GA3, GA + PAC, PAC, and control were cut into about 50-µm sections manually. Hypocotyl and stem cross-sections were stained with 0.025% toluidine blue or 5% phloroglucinol–HCl.

The diameter of the hypocotyl (μm) and the number of the xylem cell were measured from sections stained using toluidine blue; the ratio of xylem area to total area was measured from sections stained with phloroglucinol-HCl for each genotype using ImageJ software (Available online: http://rsbweb.nih.gov/ij/), in triplicate.

### 3.4. Gene Selection and Relative Expression of the SCW Genes by qRT-PCR

Birch SCW genes were selected using DGE analysis of birch transcriptome data [25]. The abundance of each gene was determined by calculating the reads per kb per million reads (RPKM) as described by the previous method [50]. GA oxidase genes were selected from the birch genome (Available online: http://birch.genomics.cn/).

Total RNA from the first to the fourth internode of each birch stem was extracted using the cetyltrimethylammonium bromide (CTAB) method. For real-time quantitative PCR analysis, total RNA was treated with DNase I and used for first-strand cDNA synthesis using PrimeScript™ RT reagent Kit (TaKaRa, Osaka, Japan). Real-time quantitative RT-PCR was performed with SYBR Premix Ex TaqTM, and primers were designed by primer 5 software (Premier, Palo Alto, CA, USA) (Table S1).

*NAC*, *MYB*, *CESA* and *PAL* genes from the birch tension wood (TW), opposite wood (OW) and normal wood (NW) transcriptome [25], and *GA20ox* genes from the birch genome (Available online: http://birch.genomics.cn/) were subjected to RT-PCR analysis under the following conditions: 94 °C, 30 s; 45 cycles at 94 °C for 12 s, 58 °C for 30 s, 72 °C for 45 s; 79 °C, 1 s for plate reading. A melting curve was generated for each sample to assess the purity of the amplified products. The relative mRNA levels were determined by normalizing the PCR threshold cycle number of each gene to that of *α-tubulin* (GenBank number: FG067376) and *ubiquitin* (GenBank number: FG065618) reference genes. Expression levels were calculated from the threshold cycle using the delta-delta CT method [51]. Three biological replicates were used in the experiments.

### 3.5. Statistical Analysis

Analysis of variance (ANOVA) of germination percentage, germination energy, germination time, hypocotyl height, fresh weight, stem height, the diameter of hypocotyl, the ratio of xylem area to total area of birch seedlings, and the relative expression of genes using qRT-PCR was performed using SPSS software (SPSS, Chicago, IL, USA). DUNCAN method was used to perform differential analyses by comparing these experiments. The level of significance was set at *p* < 0.05.

## 4. Conclusions

The effect of GA_3_ on germination of seeds, xylem development, and secondary wall biosynthesis related genes expression in *B. platyphylla* was studied. GA_3_ treatment increased the germination percentage, germination energy and germination time of seeds, hypocotyl height, and fresh weight of seedlings. Transverse sections of hypocotyls of germinated seed and stems of seedlings showed that GA_3_ increased xylem development while PAC suppressed it. Application of GA_3_ entirely ameliorated the phenotype of PAC treatment. GA_3_ treatment induced the expression of MYB and NAC transcription factors, *CESA*, *PAL*, and *GA* oxidase genes. These results suggest that birch seed germination and xylem development is promoted by GA_3_. Birch SCW genes are induced by GA_3_ and facilitate xylem development in response to GA_3_.

## Supplementary Materials

Supplementary materials can be found at http://www.mdpi.com/1422-0067/16/09/22960/s1.

## Abbreviations

GA: gibberellic acid; PAC: paclobutrazol; CESA: cellulose synthase; PAL: phenylalanine ammonialyase; SCW genes: secondary cell wall biosynthetic genes; SND: secondary wall-associated NAC domain; SWNs: secondary wall NAC domain transcription factors; NST: NAC secondary wall thickening-promoting factor.
