# Supplementary material for: Exogenous GA3 Application Enhances Xylem Development and Induces the Expression of Secondary Wall Biosynthesis Related Genes in Betula platyphylla
Source: Int J Mol Sci. 2015 Sep 23;16(9):22960–75. doi: 10.3390/ijms160922960 (PMC4613346; doi:10.3390/ijms160922960)
Supplement: Supplementary file 1 [file ijms-16-22960-s001.pdf]

Supplementary Information

Table S1. Primers used in RT-PCR analysis.

Gene Name	Primer Sequence (5′-3′)	Amplicon Sizes (bp)
BplNAC1	Forward primer: CAAACCCAACCGAGACAATG<br>Reverse primer: TGAGTGGTGCTTGCCCTATT	158
BplNAC2	Forward primer: GATAGGCTTGTGGCTTGTC<br>Reverse primer: CGATTTCGCTGTTGTAGTCC	175
BplNAC3	Forward primer: AGTGGGAACCTTGGGAGTT<br>Reverse primer: TCACATTGTTGGTGCGTCT	177
BplNAC4	Forward primer: CTGCTGCTGGATTCTGGAAG<br>Reverse primer: TCAGTTGTTTGGAGGCGAT	160
BplMYB4	Forward primer: CCCGAAGAGGACGAGATGCT<br>Reverse primer: TGGTATGGGCCTTGACGATG	193
BplMYB5	Forward primer: GAACAGTGGTGGGTGAGGAT<br>Reverse primer: TCCGAGGAAATCAATGAAGG	158
BplMYB83	Forward primer: AGCCAATAAGCGAAGTAATC<br>Reverse primer: TCTGTTCTTGAAGCCTCTGT	181
BplMYB103	Forward primer: TGCTTTCCCGGCATCTATGT<br>Reverse primer: TCTGATTTCCTTCGCACCCT	187
BplMYB85	Forward primer: CGATAGGGTTTCTTTGGACT<br>Reverse primer: AGGAGGAGGGTAGCAACAGG	150
BplCESA4	Forward primer: GATTCTTGACCAGTTCCCT<br>Reverse primer: AGTTATGATTGGCGGTTCCT	160
BplCESA7	Forward primer: CTCCCTGCTATCTGCTTGCT<br>Reverse primer: TTGCTCGTTTCTCCACCATT	162
BplPAL3	Forward primer: TTTCGAGGCCAACATACAAG<br>Reverse primer: TTCACATAAGCACTCCCATC	177
BplPAL4	Forward primer: TTTCAAGCGGATTCCAGTAT<br>Reverse primer: TGCTCCAGTGAGAAGCGTAG	184
BplGA20ox1	Forward primer: TTTCCGCTACTCTGCTGATC<br>Reverse primer: TATTCCCTGAAATGGGCTCT	192
BplGA20ox3	Forward primer: GGTGCTCTATAATGCGATGC<br>Reverse primer: GTCTGCCATTTGTTGTTTGC	154
